# Bio-Conjugated Quantum Dots for Cancer Research: Detection and Imaging

**DOI:** 10.3389/fonc.2021.749970

**Published:** 2021-10-22

**Authors:** Zhengyan Liang, Muhammad Babar Khawar, Jingyan Liang, Haibo Sun

**Affiliations:** ^1^ Institute of Translational Medicine, Medical College, Yangzhou University, Yangzhou, China; ^2^ Jiangsu Key Laboratory of Experimental & Translational Non-Coding RNA Research Yangzhou, Yangzhou, China; ^3^ Molecular Medicine and Cancer Therapeutics Lab, Department of Zoology, Faculty of Sciences, University of Central Punjab, Lahore, Pakistan

**Keywords:** QDs, cancer, bio-imaging, detection, fluorescence

## Abstract

Ultrasound, computed tomography, magnetic resonance, and gamma scintigraphy-based detection and bio-imaging technologies have achieved outstanding breakthroughs in recent years. However, these technologies still encounter several limitations such as insufficient sensitivity, specificity and security that limit their applications in cancer detection and bio-imaging. The semiconductor quantum dots (QDs) are a kind of newly developed fluorescent nanoparticles that have superior fluorescence intensity, strong resistance to photo-bleaching, size-tunable light emission and could produce multiple fluorescent colors under single-source excitation. Furthermore, QDs have optimal surface to link with multiple targets such as antibodies, peptides, and several other small molecules. Thus, QDs might serve as potential, more sensitive and specific methods of detection than conventional methods applied in cancer molecular targeting and bio-imaging. However, many challenges such as cytotoxicity and nonspecific uptake still exist limiting their wider applications. In the present review, we aim to summarize the current applications and challenges of QDs in cancer research mainly focusing on tumor detection, bio-imaging, and provides opinions on how to address these challenges.

## Introduction

Cancer is one of the most serious health threats globally. Although tremendous progress have been made in cancer diagnosis, detection, and therapy, the survivals of patients remained poor over decades (1). Cancer detection and bio-imaging are crucial clinical tools to explore the primary tumor, determine appropriate cancer therapeutic options, and evaluate the curative effects and recurrence. Currently, X-ray, computed tomography, ultrasound, radionuclide imaging, and MRI are being used for the detection and imaging of tumors. However, almost all of these techniques have their own limitations. For instance, they do not have sufficient sensitivity to detect primary or metastatic sites with small number of malignant cells. Similarly, these imaging techniques are unable to detect specific cancer surface biomarkers. Moreover, they are hazardous to humans to varying degrees. Thus, development of new techniques with high sensitivity, specificity and less hazards are urgently required.

**Figure 1:**
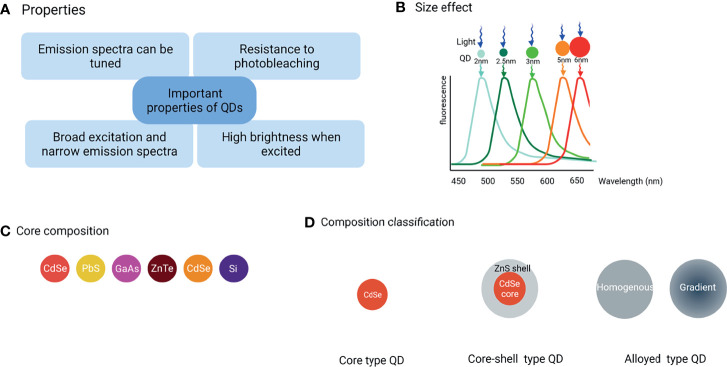
Classification of QDs and their main properties. **(A)** The important properties of QDs, **(B)** Under single-source excitation, a range of well-separated hues can be achieved by adjusting the size and shape of QDs, **(C)** The commonly used core composition of QDs, **(D)** Three major QD types are classified by composition and structure.

Recently, nanotechnology is being utilized in various fields including medicine, chemistry, and several others. QDs, often described as “artificial atoms”, are a hot topic in nanotechnology. Alexey Ekimov originally discovered QDs in a glass matrix in 1981. Four years later, the first colloidal semiconductor nanocrystallite solution was synthesized by Louis Brus. Mark Arthur Reed coined the term “quantum dots” in 1998 (2) for demonstrating the photoluminescent nanostructure that has fully quantized energy states. Consequently, many researchers began to evaluate the potential applications of QDs, especially in the diagnostics (3) because of their excellent optical and electronic properties such as superior fluorescence intensity, strong resistance to photo-bleaching, size-tunable light emission, and multiple fluorescent colors emission under single-source excitation (**Figure 1A**). These properties make them the better fluorophores than conventional fluorophores such as organic dye and fluorescent proteins. Furthermore, QDs broad absorption profiles allow simultaneous excitation of an unlimited number of well-separated colors and are excitable by a single wavelength. In addition, the emission wavelengths can be continuously tuned and precisely controlled by the size and shape during the synthesis process (**Figure 1B**). Such multicolor QDs-based probes are being utilized to analyze multiple molecular targets simultaneously. This characteristic is very beneficial in confocal microscopy to perform nanometer-resolution co-localization of multicolor QDs, in addition to reducing the amount of slices of tissue that must be cut for biomarker analysis (4, 5).

QDs have optimal surface chemistry to link with targets such as antibodies, peptides, or small molecules, producing versatile probes for biomedical applications. One of such applications is in cancer detection and bio-imaging. Cancer imaging has taken a considerable leap when Gao et al. injected PEG-coated QDs functionalized with antibodies to prostate-specific membrane antigen intravenously for the first time (6). Moreover, the resistance to bleaching over long periods allows the acquisition of sharp and well contrasted images which are especially useful for 3D optical sectioning of tumor together with its surrounding environment where bleaching of fluorophores compromises the correct reconstruction of 3D structures. Furthermore, long-term stability, high brightness, wider and continuous excitation spectrum, and deep penetration all make them ideally suitable for *in vivo* cancer diagnosis and bio-imaging.

In this review, we have discussed the fundamental, classic, and representative examples about QDs-based detection and bio-imaging in various tumors by taking in account the recent developments in this field. We have also highlighted the current challenges and proposed the best possible solutions and recommendations. We hope that this review will provide insights to inspire novel exciting discoveries to exploit QDs potential in cancer detection and bio-imaging.

## Classification of QDs

QDs composed of groups II-VI, III–V, or IV elements (**Figure 1C**) are classified into core-type, core-shell, and alloyed QDs (**Figure 1D**) by their chemical composition.

### Core-Type QDs

The CdX (X=Se, S, or Te) QDs are the most investigated QDs. However, the leaked cadmium ions are culprits for the observed cytotoxicity of cadmium-based QDs that hamper their further practical applications. However, with the emerging demand for more biocompatible QDs as the signal reporter, heavy metal-free QDs were developed such as group IV QDs including carbon-based QDs and silicon or germanium QDs (See **Figure 1D** for the core-type QDs).

### Core-Shell QDs

Core-shell QDs are the second-generation products that have been widely used as a way to adjust the photophysical properties of simple QDs. Their shell is designed carefully to enhance simple QDs photostability and photoluminescence efficiency by several folds. The core and the shell are typically composed of type II-VI, IV-VI, and III–V semiconductors with configurations such as (CdS) ZnS, (CdSe) ZnS, (CdSe) CdS, and (InAs) CdSe (See **Figure 1D** for the core-shell type QDs).

### Alloying QDs

Alloying QDs are one of the hot topics in QDs research. Alloyed QDs such as cadmium selenium sulfide (CdSeS) with both homogeneous and gradient internal structures are the newest generation of highly luminescent QDs. The homogeneous QDs have a uniform internal structure, thus the composition is same everywhere on a single QD, while in gradient QDs, alloy compositions are varied radially which means that the ratio of the first semiconductor and the second semiconductor changes gradually from the core to the surface in a gradient internal structure (7). Gradient and homogeneous alloyed QDs have varied optical and electrical properties due to their different structures, hence the internal structure of alloyed QDs is an important parameter in their applications (See **Figure 1D** for the alloying type QDs).

## QDs Functionalization

In the past few years, many efforts have been devoted to design the ideal fluorescent QDs with better biocompatibility, high photostability, uniform size distribution, abundant surface functional groups, and slow release of iron. Surface changes electrostatically and covalently, either directly or *via* a bridge, are used to functionalize QDs. For example, QDs water solubility could be increased by using a shell of functionalized silica, phospholipid micelles, and linkers such mercaptoacetic acid, dihydrolipoic acid, or amphiphilic polymers. Similarly, reactive functional groups such as amines, carboxylic acids, alcohols, and thiols can provide stability and facilitate their covalent conjugation with a variety of compounds resulting in multipotent probes. Collectively, these ligands play a critical role in making QDs more effective and biocompatible for prospective diagnostic and therapeutic applications.

### Ligand Exchange

**Figure 2:**
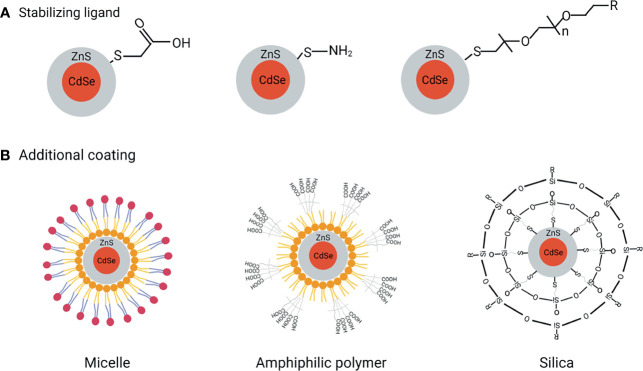
QDs functionalization to reach excellent biological applications. **(A)** Ligand exchange to improve QDs stabilization, **(B)** Additional coating methods improve water dispersibility, biocompatibility, and bioconjugation.

The ligand exchange process is simply defined as the substitution of a functional ligand for a nonfunctional ligand in order to provide QDs additional features such as solubility and stability (**Figure 2A**). Thiols (-SH), carboxyl (-COOH), and PEG are the most commonly used ligands. Thiol groups are frequently utilized as anchoring groups that bind to the surface of QDs, while carboxyl groups are frequently used as hydrophilic endings that provide extra stability, and PEG is frequently used to improve QDs solubility range from pH 5 to 12 for more than a year (8). However, there are a number of drawbacks of this method. For instance, in aqueous environments, the thiol molecules may form disulfides and detach from the surface, producing QDs aggregation and oxidation, and the surface change may result in an irreversible drop in QDs quantum yield.

### Silanization

Silanization, which involves the insertion of a silica shell over the QDs, is an effective covalent coating approach for modifying hydroxyl-rich material surfaces (**Figure 2B**). The main advantage of silanization is that the ligand molecules are highly cross-linked and form a chemically stable capping agent. Besides improving the biocompatibility, the end terminal groups of the silane shell can expose either their thiol, phosphonate, or methyl terminal ends for further coating of QDs. Moreover, silanization is also preferred because of less toxicity compared to other ligands (9). Finally yet importantly, QDs response to light can be finely tuned by controlling the thickness of the silica shell. CdSe/CdS/ZnS-QDs, for example, have been encapsulated in silica nanoparticles using a water-in-oil reverse microemulsion method (10). To test their applicability, the silica-coated QDs are next modified with amino, carboxyl, and epoxy groups and stabilized with polyethylene glycol (PEG) fragments. These modified QDs were found efficiently conjugated with antibodies and applied as a fluorescent label in the immunoassay detection (11).

### Encapsulation by Amphiphilic Ligands

Amphiphilic polymers are used to provide additional stability and flexibility to QDs under complex biological conditions. Poly (acrylic acid) copolymer, the highly charged linear polyelectrolytes containing carboxylic acid groups, are a class of amphiphilic polymers. Abdolahi and colleagues have reported the fabrication of a starch-g-poly (acrylic acid)/ZnSe-QDs hydrogel that may act as a dye adsorbent and photocatalyst (12). This approach has received much attention in designing efficient photocatalysts considering the high stability of QDs in the hydrogel system (**Figure 2B**). Phospholipids are another type of amphiphilic polymer with a polar and nonpolar portion in the structure that impart great biocompatibility and amphiphilicity in function. Besides, they also have a good emulsifying property that can stabilize the emulsions (**Figure 2B**). These unique features make phospholipids most appropriate for biological applications. For instance, *in vitro* and *in vivo* imaging by QDs encapsulated with phospholipid micelles was described by Dubertret et al. (13). This QD-based probes performed as *in vitro* fluorescent probes when coupled with DNA, hybridizing with particular complementary sequences, and showed to be stable, nontoxic, cell-autonomous, and photobleached slowly when injected into Xenopus embryos.

### Microsphere/Microbead Coatings

Incorporating QDs in microspheres or microbeads is one of the great interests in biological applications. For example, Liu et al. designed the QD-microsphere-based immunochromatographic quantitative ciprofloxacin (CIP) test strips (14). The QD-monoclonal antibody probes adhered to CIP and were unable to be caught by the CIP-bovine serum albumin conjugation dispersed along the T lines, resulting in reduced fluorescence intensities. These test strips provide a low detection limit and a wide linear detection range with high sensitivity and accuracy along with good selectivity, reproducibility, and stability which might be used in rapid on-site testing.

## QDs for *In Vitro* Diagnosis and Imaging

**Figure 3:**
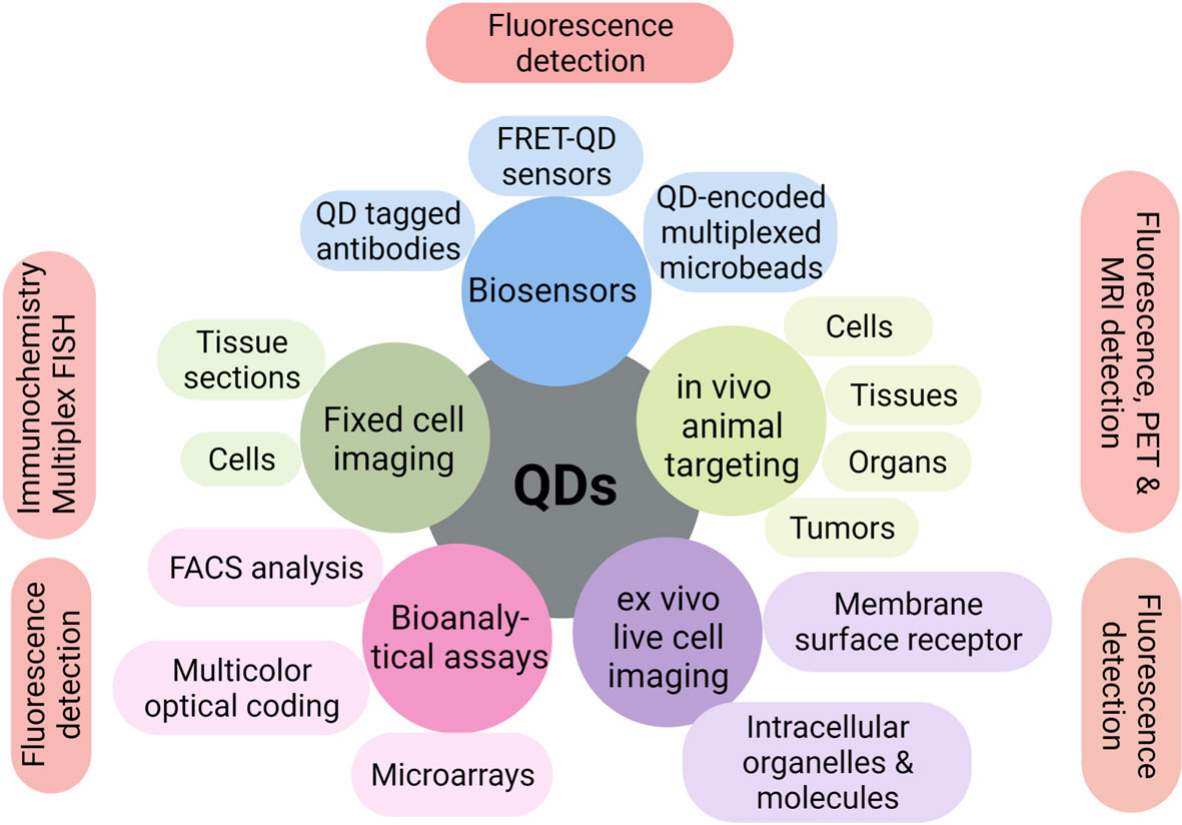
Multiple applications of QDs in biotechnology. QDs have shown excellent multiple applications in biosensors, bioanalytical assays, fixed or live-cell imaging, and *in vitro* or *in vivo* imaging over the past few years.

Excellent properties of QDs make them superior to traditional fluorescent organic dyes. QDs-based signal probes are of great interest and have been tested in numerous biotechnological applications. Some of the early and most successful uses of QDs have been in immunofluorescence labeling of fixed cells and tissues (15, 16), immuno-staining of membrane proteins (17, 18), microtubules (19), nuclear antigens (20, 21), and fluorescence *in situ* hybridization of chromosomes or combed DNA (22, 23) (**Figure 3**).

Overexpressed receptors play a crucial role in target identification and bioimaging in many malignancies. QDs conjugated with cancer-specific ligands/antibodies/peptides are very effective in detecting and imaging human cancer cells derived from prostate cancer (6), breast cancer (24), pancreatic cancer (25), metastatic tumor (26), glioblastoma (27), cancers of bone marrow (28), and tongue (24). **Table 1** (29–39) lists some of the common receptors targeted by QDs in *in vitro* studies, and these QDs-based probes are divided into antibody-based and ligand-based categories based on the functional groups attached to the surface of QDs.

**Table 1:** Selected reports on applications of QDs in cancer diagnosis.

Targeted receptor	QDs	Cell culture
Integrin α_v_β_3_	PEGylated CdTe; Polymer-coated CdSe/ZnS	Human glioblastoma cells (U87MG cells), Human oral squamous carcinoma cells (BcaCDE885);
Folate receptor	NAC capped and alloyed with CdTeS	Bel-7402 human hepatoma cells that overexpress folate (FR+) and A549 human lung cells with low expression of folate receptor (FR-);
Transferrin	Protein-coated alloyed ZnHgSe	Hela cells, MDA-MB-435 human breast cancer cells;
VEGFR	CdSe/ZnS coated with oleylamine poly (aspartate)-graft-PEG-dodecylamine	Human liver cancer (HepG2) cells;
TAG-72	CdTe QDs and Fe3O4 NPs; CdTe/MPA QDs	Colon carcinoma cells LS174; Gastric cancer cell line MGC80-3;
HER-2/neu	PEGylated; PEG-coated QD800	Human breast carcinoma cells (SKBR-3 and KPL-4);
MMP-2	CdTe QDs	MCF-7 cells;
Glycans	Mercapto-succinic acid-coated CdTe QDs	Fibroadenoma and ductal carcinoma;
Mucin 1 protein	Magnetic and CdTe QDs immobilized on SiO2	MCF-7 cells;

NAC, N-acetyl-L-cysteine; AOM, Azoxymethane; VEGFR Vascular endothelial growth factor receptor; MMP-2, Matrix metalloproteinase-2; TAG-72, Tumor-associated antigen glycoprotein 72.

On the one hand, QDs have been widely used for prolonged fluorescent visualization by conjugating with antibodies (primary or secondary) against overexpressed receptors on cancer cells. For example, Han et al. designed *in situ* automatic DNA assembly reaction and applied it for the simultaneous identification of dual targets using QDs-based probes (40). Chained strand displacement events are triggered after the capture probes detect the surface biomarkers epidermal growth factor receptor (EGFR) and intercellular adhesion molecule-1 (ICAM-1) in the triple-negative breast cancer cell MDA-MB-231. Then, utilizing QDs as electrochemical probes, increased electrochemical signaling was established to disclose the co-expression of the two targets. Mirzababaei M et al. bounded NL2 peptide to the surface of poly 3,4-dihydroxy-L-phenylalanine (Poly L-DOPA) graphene quantum dots (GQDs) and imprinted by doxorubicin (DOX) (41). The nanoprobes linked to SK-BR-3 cells can be observed due to the presence of GQD particles, and DOX is released in the tumor cells.

As anticancer antibodies are quite expensive, many researchers investigated alternative ligand-based QDs including folic acid (FA), epidermal growth factors, transferrin, and a few aptamers to target cancer cells. For instance, Qi et al. developed GQDs that suppress the growth of the tumor by selectively damaging the DNA of the cancer cells (42). The nucleus-targeting TAT peptides (TAT-NGs) were added to the amine-functionalized GQDs, which were then grafted with cancer-cell-targeting FA modified PEG *via* disulfide linkage (FAPEG-TNGs). These FAPEG-TNGs exhibited good biocompatibility, nuclear uptaking, and cancer cell targeting. Furthermore, Singh G et al. conjugated fluorescent CdSe/CdS/ZnS and CdTe QDs stabilized with 3-mercaptopropionic acid (MPA) and mercaptosuccinic acid (MSA) with FA, which showed higher cellular internalization (43). Similarly, Salova AV et al. constructed EGF-QDs complex by the biotin-streptavidin system (bEGF-savQDs) that can enter Hela cells *via* temperature-dependent clathrin-mediated EGFR specific pathway (43).

## QDs for *In Vivo* Diagnosis and Imaging

The basic principles underlying *in vitro* can also be applied to *in vivo* diagnosis and imaging of cancer cells. Functional *in vivo* QDs can be produced by conjugating to antibodies, biotin, aptamers, or other biomolecules. However, there are several challenges compared to *in vitro* applications such as the penetration depths of excitation light, tissue autofluorescence, toxicity, and pharmacokinetics. Collectively, under *in vivo* conditions, QDs-based probes must be able to emit stronger fluorescence, superior photostability, shield luminescent cores from leaking, and have more functional groups. Furthermore, QDs should be monodisperse to provide uniform fluorescence of QD-labeled targets, modest in size to preserve the targeted molecules’ natural characteristics, and low in nonspecific adsorption (44, 45).

### QDs for *In Vivo* Diagnosis

**Figure 4:**
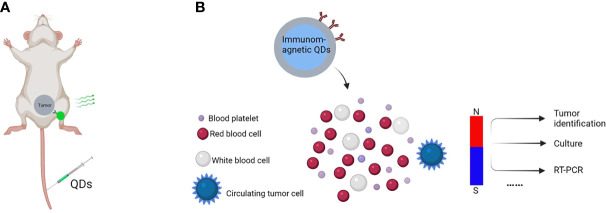
*In vivo* tumor imaging and CTCs detection. Schematic of *in vivo* tumor imaging and CTCs detection, **(A)** QDs-antibody conjugates were injected through the tail vein that distributed efficiently and uniformly in the tumor due to specific binding between cancer cell surface antigen and QDs-antibody conjugates, **(B)** QDs-antibody conjugates with fluorescent and magnetic properties are used to detect CTCs in the bloodstreams of animals with no disruption to their function.

In mouse models, QDs coupled with several cancer indicators have been examined *in vivo.* For instance, Gao et al. were the first to apply QDs-antibody conjugates *in vivo* (6) (**Figure 4A**). They administered QDs-PSMA antibody systemically to a mouse bearing subcutaneous human prostate cancer. The QDs-antibody conjugates distributed efficiently and uniformly in the prostate tumor due to the specific binding between PSMA antigen in prostate cancer cells and QDs-PSMA antibody conjugates. Similarly, Liu et al. introduced a nanosystem that allows selective background quenching to gain exceptional tumor-specific fluorescent signals (46). This system uses near-infrared QDs and a membrane-impermeable etchant as a cation donor. Briefly, QDs were delivered intravenously into orthotopic breast and pancreas tumors in mice using the tumor-penetrating iRGD peptide. Subsequently, etching quenches excess QDs, leaving intact QDs in the extravascular tumor cells to deliver a highly tumor-specific signal and facilitating the renal clearance of metal ions released from QDs.

Apart from that, QDs can also detect multiple cancer markers such as circulating tumor cells (CTCs) and multiple biomarkers with more sensitivity than standard immunohistochemistry (47, 48). CTCs are a kind of tumor cells playing vital roles in cancer metastases, as their capture, isolation, and genetic profiling are meaningful for the early diagnosis and control of metastatic. Nondestructive and sensitive detection is essential for learning more about CTCs. For example, Kuo et al. used antibody (anti-CD24 or anti-CD133) coupled QDs to image serum CTCs in real time in living mice (49). They developed a noninvasive cancer model by injecting pancreatic cancer cells containing fluorescent proteins into the earlobes of mice. CTCs with fluorescent proteins in the bloodstream may be seen consistently after breaking off from the solid tumor. In another study, QDs were employed for CTCs “omics” (50, 51). In 90% of cases, CTCs could be trapped individually, and the expression level of protein biomarkers on a single CTC could be measured (52). As showed in **Figure 4B**, a typical model has been used for detecting CTCs.

**Figure 5:**
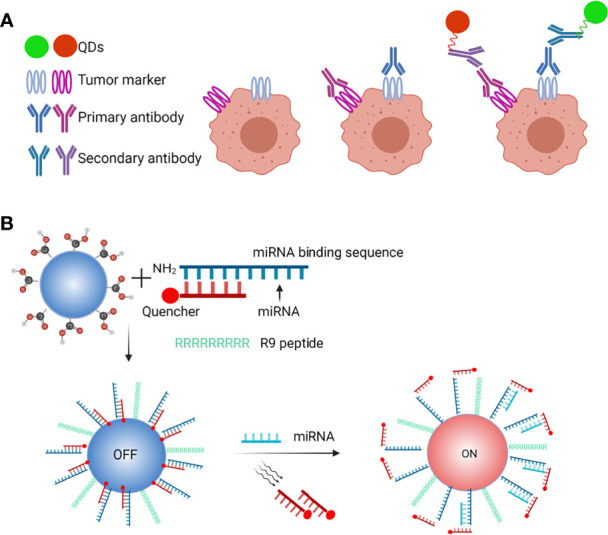
QDs-based probes in microRNA and multiplexed biomarkers detection. **(A)** Schematic of QDs-based microRNA nanosensor, **(B)** QDs for facile and simultaneous detection of multiple biomarkers.

Because various emission wavelengths activated by a single light source may be created by altering chemical composition and size, QDs offer considerable benefits in multiplexed diagnostic detection (**Figure 5A**). Multiple tumor markers in clinical samples can be diagnosed simultaneously with great accuracy and consistency by combining QDs-encoded microbeads with flow cytometry (53). Guo et al. constructed a multiplex electrochemiluminescence (ECL) immunoassay for simultaneous assessment of two unique tumour markers, alpha-fetoprotein (AFP) and carcinoembryonic antigen (CEA), using multicolor QDs as labels and graphene as a conducting bridge (CEA) (54). A standard sandwich immune complex was established on the glass carbon electrode to obtain recognizable ECL signals, with QDs525 and QDs625 tagged on secondary anti-AFP and anti-CEA antibodies, respectively. With a working range of 0.001-0.1 pg/ml and no noticeable cross-reactivity, the multiplex ECL-immunoassay allowed simultaneous monitoring of AFP and CEA in a single run. This immunoassay provides a simple, sensitive, specific, and repeatable approach for simultaneously detecting tumor markers in clinical situations. Qu et al. used three QDs-encoded microbeads (Qbeads) to simultaneously identify three miRNA biomarkers, miRNA-21, miRNA-221, and miRNA-16, in ~500 human hepatoma cancer cells (55). These tests can be completed in a single step, resulting in low cost and simple operation. However, the majority of the biosensors described thus far can only perform double or triple analysis.

Collectively, these approaches not only identify multiple biomarkers or CTCs but also assess circulating microRNAs (56). As shown in **Figure 5B**, QDs have been applied in a model of circulating miRNAs detection and exosomes to acquire integrated information (57).

### QDs for *In Vivo* Imaging

QDs have a lot of potential in cancer bio-imaging because of their brilliant fluorescent signals and multiplex capabilities. They have a lot of selectivity and sensitivity in detecting early-stage tumors and their metastases. Akerman et al. originally investigated the application of QDs for *in vivo* imaging (58). They discovered that CdSe/ZnS QDs coated with peptides were preferentially distributed among endothelial cells in the lung blood vessels after injecting them into mice tail veins. However, QDs’ efficiency is severely limited by tissue autofluorescence, absorption, and photon scattering. Thus, to separate tissue autofluorescence from QD signal in transplanted malignancies, a spectral demixing technique must be created. Kim et al. were the first to address this issue by injecting NIR-emitting QDs intradermally into living mice and pigs to improve the signal-to-noise ratio by reducing photon scattering and penetrating deeper into tissues (59). Since then, QD-based immunohistochemistry combined with NIR-I (650-950nm) and NIR-II (1000-1350nm) fluorescence imaging has been widely used in personalized oncology to assess tumor origin and progression.

In recent years, many heavy element-free QDs with emission in the NIR-I and NIR-II windows have been developed. Ag2X (X=S, Se, and Te) QDs are great candidates since they are brilliant and photostable (60, 61). After biofunctionalization, these QDs could be used for *in vivo* fluorescent tumor detection and imaging (62–65). NIR QDs with various components have also been synthesized for bio-imaging, including dazzling CuInS2/ZnS QDs with tunable emission from 750 to 1100 nm (66), and 1200nm emitting PbS/CdS/ZnS QDs (67). However, developing NIR-II QDs with a high photoluminescence quantum yield (PLQY) and good biocompatibility is difficult. In a mouse model using multiplexed lymph node imaging, Saeboe et al. reported the reddest emitting indium phosphide quantum dots (InPQDs) to date. In the first optical tissue window, they exhibited tunable NIR photoluminescence (PL) as well as PL multiplexing while avoiding hazardous components (68). By widening the range of controllable direct-bandgap emission from InP-based nanostructures, these nanoparticles efficiently overcome a synthetic barrier that has stopped InPQDs from reaching their full potential. Many technologies have been employed to give real-time cancer imaging *in vivo*, as illustrated in **Table 2** (58, 69–73). Overall, such high-quality NIR QDs could provide unrivalled sensitivity, speed, and real-time dynamic *in vivo* imaging.

**Table 2:** Preclinical use of NIR biocompatible QDs in cancer imaging.

Imaging technique	QDs	Studies/animal model
NIR-optical imaging	CuInS2/ZnS	SLN imaging in mice, Bel-7402 tumor imaging, Panc-1 tumor imaging, Brain glioblastoma tumor;
	AgInSe2/ZnS	Active targeting to ayb3 integrin receptor overexpressed ayb3-positive MDA-MB-231breast cancer;
NIR- optical imaging	Ag2S	Passive targeting to 4T1 tumor through EPR effect, Deep tissue imaging the liver, spleen, and blood vessels of the whole mouse;

High spatial and temporal resolutions, 3D tomography, a high signal-to-noise ratio, and noninvasiveness are all sought for in modern imaging technologies, whether for basic research or biomedical applications. However, owing of differences in biodistribution and other pharmacologic properties, a single imaging method cannot address all of these needs, and separate deployment of numerous imaging probes with diverse modalities is not an appropriate solution either. As a result, in order to achieve multimodal imaging technology, it has been attempted to unite the features of many imaging modalities in the same chemical entity. Because they can integrate a variety of probe properties, QDs are one of the most exciting multimodal probes. When fluorescence imaging with QDs is combined with molecules/materials that exhibit paramagnetism and radioactivity, for example, MRI and radiography imaging can be combined (74). MRI-fluorescence imaging is one type of bimodal imaging using QD probes, which has the advantage of being durable in multimodal imaging. Mulder et al. looked into multifunctional CdSe/ZnS QD probes as an example of QD-based MR-fluorescence bimodal imaging. They used their multifunctional probe to successfully target endothelial cells, which was detected using both fluorescence and MRI imaging (75). Mn-doped QDs particularly Mn-doped ZnSe QDs are another type of QDs-based MR-fluorescence bimodal imaging with a quite low concentration of dopant (Mn) (76). To achieve fluorescent/MRI dual-modal bio-imaging *in vivo*, Mn-doped ZnSe QDs were loaded into pores of mesoporous silica nanoparticles. Similarly, magnetically tailored Cd-free CulnS2 @ ZnS: Mn QDs were developed as potential dual-modality probes for fluorescence and MRI imaging of malignancies *in vivo* and found to be effective against both subcutaneous and intraperitoneal tumors (77). Individual imaging technology has advanced to the point that biomedical cancer imaging could gain a new dimension and momentum with the design and synthesis of appropriate multimodal probes based on QDs that meet *in vivo* cancer imaging standards. Magneto-fluorescent GdNS@CQDs with remarkable water dispersibility, high stability, high quantum yields, and outstanding magnetic characteristics were successfully synthesized in an independent investigation, and these qualities made them suitable for dual model imaging (78). Multimodal probes appears to be realistic, given the rapid expansion of QDs and the abundance of information on the molecular basis of cancer and other illnesses.

## Challenges

Although QDs offer a lot of good qualities, there are still certain obstacles to overcome, and more tests are needed to improve their performance before they can be widely used in medical treatments.

### Cytotoxicity

The cytotoxicity of QDs is one of the major concerns that has slowed their development. Much of the research shows that this toxicity is dose-dependent (79, 80). For example, Lu et al. studied the effects of CdSe/ZnS core/shell QDs in hepatic L02 cells and observed that cytotoxicity rises with QD concentration (from 5 to 80 nM) (81). Similarly, Yang et al. found that a high dose (5 nM per rat) of QDs causes severe toxicity in lactating rats, including splenomegaly, multiple organ injuries and inflammation, endocrine disruption, and rat death, while a low dose (1 nM per rat) causes mild toxicity, including weight loss, mild hematology, serum biochemistry, and histopathological changes (82).

The cytotoxicity of QDs is mostly governed by their physiochemical characteristics, including hydrodynamic diameter (HD) size, surface properties like charge or ligand, and shape. The HD size, determined by both the inorganic core size and the surface coating, has a tremendous impact on the uptake and clearance of QDs (83, 84). Choi et al. used a rodent model to test renal filtration and urine excretion of QDs with five distinct HD diameters ranging from 4.36 to 8.65 nm (85). When the size was smaller than 5.5 nm, a rapid and efficient urinary excretion from mice was clearly observed. However, QDs with larger HD size were not captured for renal excretion. Apart from excretion, the blood half-life increased 25-fold, from 48 min to 20 h, with the HD size increase from 4.36 to 8.65 nm. The presence or lack of a shell, the surface charge, and other surface characteristics of QDs can have a variety of effects on their toxicity. The better protected the shell is, the slower the discharge of heavy metal ions, which is one source of QD toxicity. According to Kirchner et al., ZnS coating protects the QD core from oxidation, reducing Cd^2+^ leakage and cytotoxicity (86). On the other hand, drug transport studies in the kidney show that in contrast to negatively-charged QDs, positive-charged QDs readily pass through the anionic glomerular basement membrane and are rapidly excreted into urine which means less cytotoxicity exists in the body (87). Because most QDs are spherical or spheroid-like, their form isn’t an apparent influencing element. In endocytosis and phagocytosis, the shape of QDs is useful in the membrane packing process. Endocytosis of spherical QDs is quicker than that of other non-spherical nanomaterials, such as carbon nanotubes, according to certain research, which indicates less harmful consequences (88, 89).

Pharmacokinetic factors such as half-life duration, biodistribution, degration, rate and route of excretion, and others can be used to quantify the impact of the physicochemical qualities listed above on toxicity. A lot of research into the pharmacokinetics of QDs in cells and small animals have been done so far. Ballou et al. investigated the impact of surface coating on blood circulation and organ biodistribution in the first place (90). Polyacrylic acid-coated QD (PAA-QD) conjugated to low molecular weight PEG (750 Da) and intravenously injected into nude mice exhibited short blood circulation half-life (t1/2 < 12 min) with predominant uptake by the liver, spleen, lymph nodes, and bone marrow. When the same QD was decorated with PEG5000, the blood t1/2 was raised to 3 hours, with reduced uptake in the liver, spleen, and lymph nodes (90). Similar studies by other groups showed that 15-20 nm QD coated with PEG5000 exhibited long t1/2 of 5-8 h (3, 6, 91, 92). Although rapid clearance of QDs from the body may reduce the potential toxic damage influence on the organs, tissues or cells, sufficiently long blood half-life time is desired for enhancing their accumulation in the targeted site. Furthermore, in all these reports, the QD biodistribution was qualitatively determined based either on QD fluorescence in tissue sections using fluorescence and confocal microscopy or the whole body fluorescence imaging of living animals. Fischer et al. described the first quantitative biodistribution study of QD by detecting the Cd atoms in the blood and organs of rats injected intravenously with QD (93). Once QDs have been taken up by the target cells, they are mainly directed to the endosomal/lysosomal pathway where they are degraded. Some QDs disintegrate quickly, but others can linger in the body for weeks or months, causing serious harm. Previously, Ballou et al. showed that QD could be found in the liver, lymph nodes, and bone marrow of mice for up to many months (90). Frangioni and colleagues found a link between the size of the QD and the degree of elimination (85, 94). Four (4) hours after injection, QD with an average diameter of 5-6 nm, which is below the renal filtration threshold, were eliminated *via* urine. Larger QDs stayed undesirably in the liver, potentially increasing the toxicity of these nanoparticles in the long run. The fate of QD after various administration routes has also been investigated. After subcutaneous, intradermal, or intraparenchymal injection in live animals, polymer-coated QD with an average diameter of 15-20 nm were observed to move quickly to the sentinel lymph nodes (SLN) (59, 95–98). This observation of detecting SLN resident nodules, can aid in the detection of cancer metastasis. Overall, it can be observed that depending on the properties of the QD (size, surface charge, and coating) and the method of administration, QD can remain in various organs in living animals. Furthermore, these investigations have revealed that QD may accumulate in the body for long periods of time, indicating that more research is needed to determine QD’s long-term toxicity before it is used in clinical applications.

In addition, studies have also shown that QDs can cause immunotoxicity (99–105) and genotoxicity (106, 107). QDs cause apoptosis and necrosis in immune cells, and poor clearance of apoptotic cells by scavenger phagocytes may contribute to autoimmunity (108), while QD interactions with the immune response might alter immune-specific signaling pathways, resulting in alterations in immune cell function (109). There are direct and indirect pathways of QD-induced genotoxicity, according to published research (107, 110, 111). When cells absorb QDs, they may come into direct contact with genetic material, inflicting physical or chemical harm. However, the most likely mechanism of QD-induced genotoxicity is indirect, and oxidative stress is thought to be the most important indirect mechanism (112).

In conclusion, to pave the way for clinical utilization of QDs, more long-term toxicological and pharmacokinetic studies addressing QDs degradation, excretion, persistence, and immune response and precise control over the construction of QDs-based probes from the core to surface coating are needed before QDs-based probes may be declared as verified safe nanoparticles. Furthermore, more studies are needed in synthesis process to develop heavy metal free or robust covering QDs to reduce toxicity Last but not least, monitoring of the quantitative application dosage of QDs to set up safe ranges under different conditions is also urgently required.

### Nonspecific Uptake by the Reticuloendothelial System

According to all *in vivo* animal imaging investigations described thus far, a number of naked and non-targeted QDs may accumulate in the reticuloendothelial system (RES), which comprises of phagocytic cells located in the liver, spleen, lymph nodes, and bone marrow, after systemic delivery. This non-specific absorption prevents QDs from being targeted and raises concerns regarding RES toxicity.

One way to improve tumor targeting specificity is through modifying the surface of QDs to make them persist longer in the bloodstream by introducing high molecular weight PEG molecules (113). Of course, the surface coating and QDs must be stable enough to circulate for an extended period of time. For instance, mice were injected with peptide-conjugation iron oxide nanoparticles after being pre-treated with decoy liposome particles to eliminate plasma opsonin that bind to nanoparticles and prevent RES absorption. By increasing the half-life of iron oxide nanoparticles, intravenously given decoy liposomes greatly improved active targeting of xenograft breast cancers (114). Similarly, another research conjugated streptavidin-coated QDs (SA-QDs) with hydrazinonicotinamide (HYNIC) to boost QDs negative charge to suppress surface opsonization (115). When delivered intravenously to mice, HYNIC attachment exactly reduced SA-QD engulfment by macrophages, drastically enhanced SA-QD circulation, and lowered their RES absorption, according to confocal microscopy and fluorescence-activated cell sorting. Overall, surface functionalization could minimize toxicity by reducing non-specific RES phagocytosis.

### Specific Targeting to Tumor Cells

Selective tumor targeting of QDs *in vivo* is more difficult than *in vitro* for a variety of reasons. To begin with, complicated anatomical structure and physiology, such as vascular endothelium, provide challenges for QDs in tissues and organ systems. Secondly, protein-based ligands are sensitive to degradation, resulting in a loss of targeting capability, and most conjugation chemistries do not allow controlling over complex macromolecules tethered to QDs, such as antibodies, resulting in partial or entire loss of cell-binding activity. Finally, only a few cell targeting ligands are truly tumor specific, meaning that QDs bind solely to cancer cells and not to normal cells.

QDs targeting detection and imaging should be adjusted or aided by other reagents to pass through various biological barriers and reach the target areas to improve selectivity. For example, Yong et al. used permeation enhancers based on organic solvents in modest percentages to improve intracellular targeted distribution of QDs transverse intracellular obstacles, particularly vesicle entrapment (116). In another study, Li et al. demonstrated that carbon QDs functionalized with multiple paired -carboxyl and amino groups that bind to the large neutral amino acid transport 1 (LAT1), which is expressed in most tumors, selectively accumulate in human tumor xenografts in mice and in an orthotopic mouse model of human glioma with high specificity, by mimicking large amino acids (117). Furthermore, other tumor specific antigens (TSA), such as Mucin 1 (MUC1), which is absent in normal tissues but overexpressed in almost all human epithelial cell malignancies, should be investigated in QDs targeting applications.

## Perspectives

Trends in the application of QDs in cancer research clearly show that QDs can serve as powerful tools for cancer diagnosis and bio-imaging. An ideal clinical setting would be one in which the primary tumor and metastatic tumors could both be diagnosed early and efficiently treated noninvasively without the need for surgery using QDs-based probes. However, currently available electrochemical biosensors are rudimentary and unsophisticated, and practically all of the current synthesis and application are done separately. There is still a room for improvement in terms of producing QDs probes with improved target selectivity, signal intensity, multimodality, and therapeutic potential, as well as decreased cytotoxicity and non-specificity. Additionally, an integrated and pipelined platform is required to simplify the detection process and achieve higher automation before they can be widely used to diagnose and treat cancer and other disorders.

**Figure 6:**
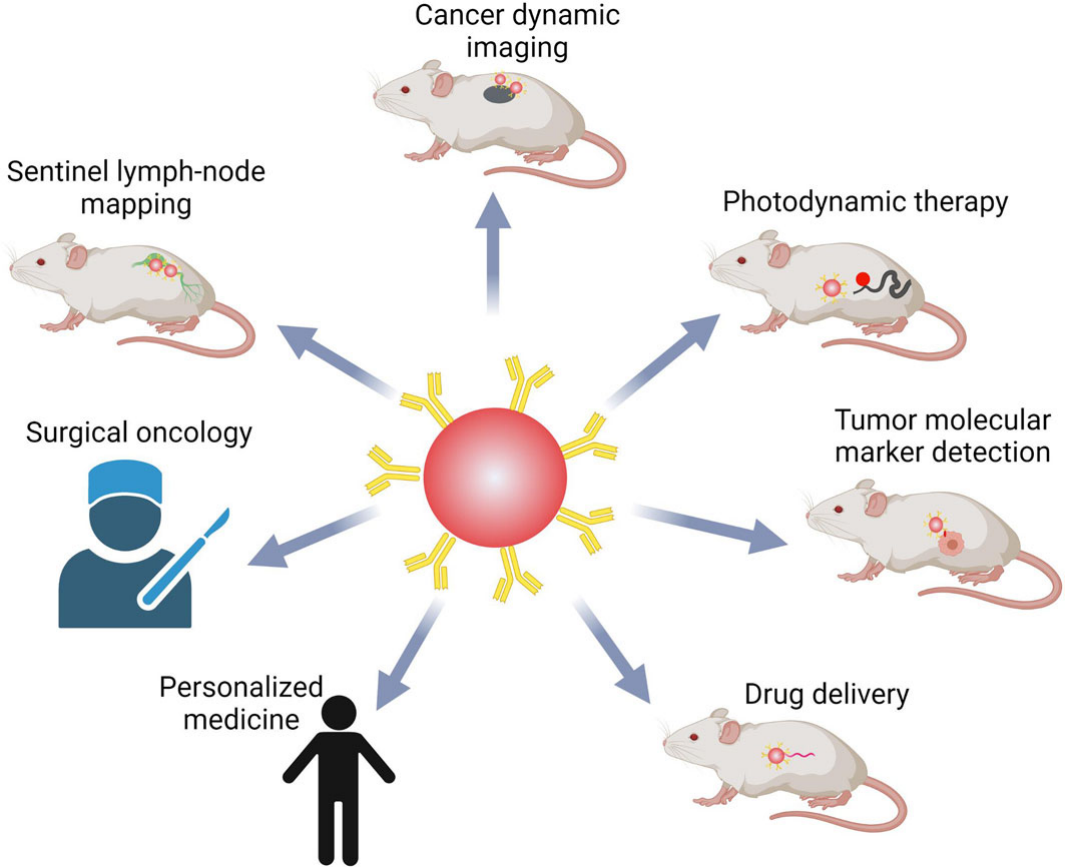
QDs-based probes in bio-imaging, bio-detection, and bio-medicine. QDs are very promising in cancer research and treatment, including real-time systemic imaging, minimal side effects, photodynamic therapy, detection of multiple biomarkers, and delivering drugs. QDs might also help with surgical oncology and individualized precision medicine.

Several aspects are listed below that may require attention in order to achieve our goal. For example, for a maltose sensor, a fluorescence-quenching molecule that can be detached or cleaved upon attachment to the target or in the presence of a chemical species or enzyme was found to generate QDs with novel features (118). This would be especially advantageous in intracellular applications, where removing unbound QDs and lowering background signal is impossible. To develop their targeting potential, new approaches for QD synthesis and structural customization are necessary. Secondly, there is still a pressing need to develop natural, mild, efficient, and less interfering QD labelling procedures, because QDs functionalization has always been a difficult problem in many biological applications, either dynamic imaging or detection. Other similar technologies, such as *in vivo* cross-linking technology pioneered for dyes, may help with QD functionalization. Several molecular evolution-derived pairings have been reported, with fusion peptides ranging in length from 200 to 30 amino acids (119, 120). Thirdly, microfluidic technology has been used in a variety of applications, including the manufacturing of QDs for their biological uses (121). Until today, researchers could split microfluidic devices into continuous-flow, segmented-flow, and droplet microfluidics, each with its own set of advantages. Continuous processing, for example, could precisely control the size of QDs (122), segmented flow microfluidics could make the size tunable and the reaction time on the millisecond scale (123), and droplet microfluidics could manufacture diverse QDs or encapsulate QDs for further use (124). Thus, to improve the quality and quantity of QDs, microfluidics technology should be studied. Finally, despite the development of multiple sensitive ways for detecting cancer biomarkers using QDs, only a few have made into clinical diagnosis. To be suitable for storage and use, the colloidal stability of functionalized QDs should be considerably improved. If the integrated and pipelined platform is accomplished, QDs might be employed not only in superior hospitals, but also in primary hospitals to satisfy the needs of more people, potentially playing a key role in cancer early detection and treatment, resulting in a higher level of health. Overall, QDs hold a lot of promise in terms of cancer molecular targeting bio-imaging and diagnostics (**Figure 6**).

## Conclusion

The rapid development of QDs technology has already fulfilled some of the hopes of developing new and more effective cancer diagnostic and imaging probes. Their properties and successful conjugation with biomolecules have made the active targeting of tumors possible. Despite the promise and usefulness of QDs in cancer detection and imaging thus far, there are still challenges to overcome in terms of boosting sensitivity, optimizing specificity, and lowering QD toxicity before clinical applications can move forward. We have only scratched the surface of QDs-based nanotechnology, and there is still a long way to go before we achieve a true breakthrough with the cooperation between researchers and professional staff in other areas. We are confident that QDs will change not only customized oncology, but also customized medicine.

## Author Contributions

ZL collected the data, draw figures, and wrote the manuscript. HS proposed the idea, modified, supervised, and approved the final version of the manuscript. MK helped to edit the manuscript. JL provided professional advices in the minor revise previously. All authors contributed to the article and approved the submitted version.

## Funding

This study was funded in part through the Startup Foundation for Advanced Talents and Science and Technology Innovation Foundation at Yangzhou University (HS).

## Conflict of Interest

The authors declare that the research was conducted in the absence of any commercial or financial relationships that could be construed as a potential conflict of interest.

## Publisher’s Note

All claims expressed in this article are solely those of the authors and do not necessarily represent those of their affiliated organizations, or those of the publisher, the editors and the reviewers. Any product that may be evaluated in this article, or claim that may be made by its manufacturer, is not guaranteed or endorsed by the publisher.
